# Supplementary figures and images for: Public interest in palliative care in Latin America: A Google Trends analysis
Source: PLoS One. 2024 Jul 18;19(7):e0306312. doi: 10.1371/journal.pone.0306312 (PMC11257273; doi:10.1371/journal.pone.0306312)

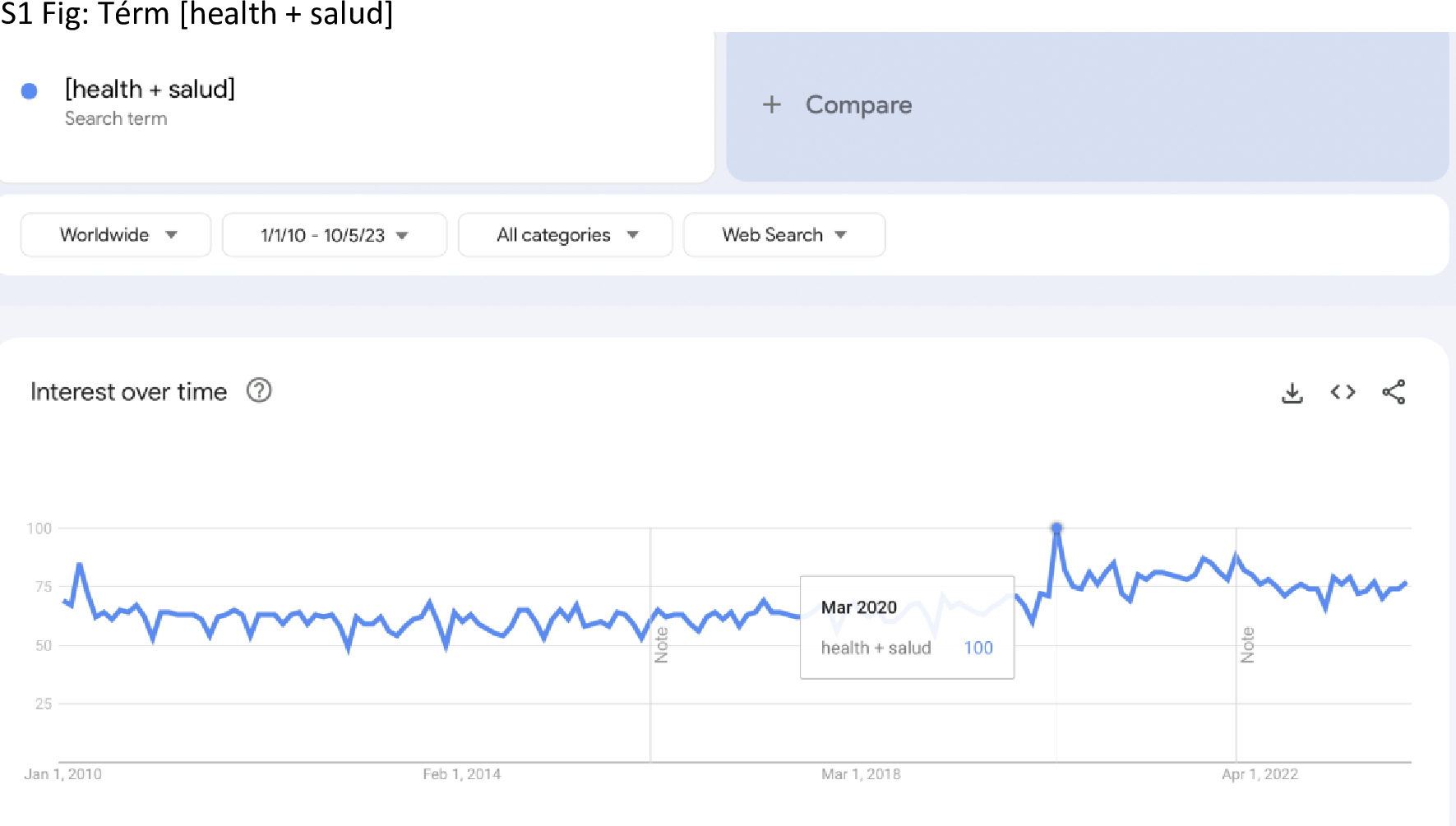

Supplement: S1 Fig — (TIF) [file pone.0306312.s001.tif]

S2 Fig: Term [palliativism] in the Portuguese language.
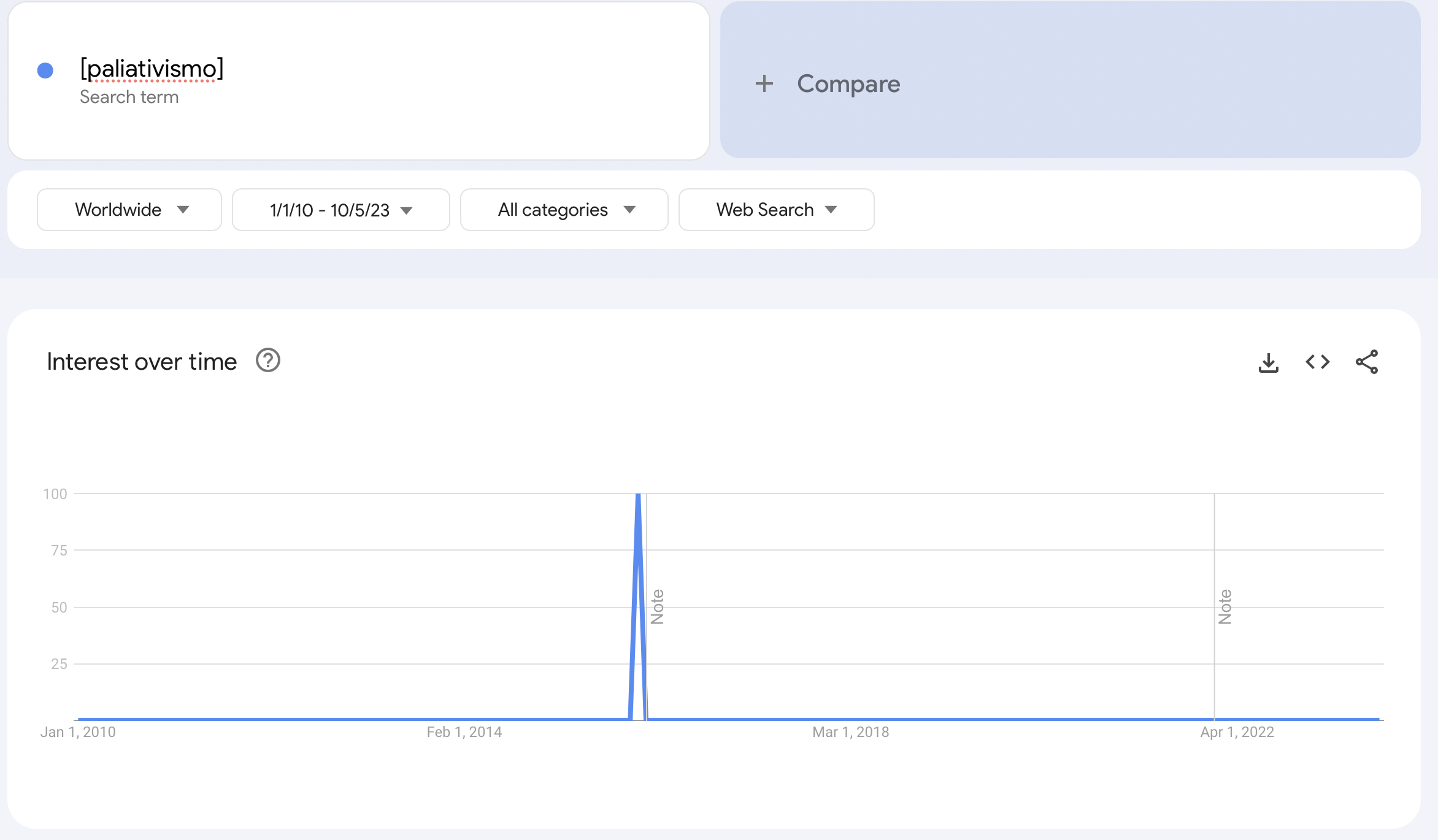

Supplement: S2 Fig — (DOCX) [file pone.0306312.s002.docx]
