# Supplementary material for: Public interest in palliative care in Latin America: A Google Trends analysis
Source: PLoS One. 2024 Jul 18;19(7):e0306312. doi: 10.1371/journal.pone.0306312 (PMC11257273; doi:10.1371/journal.pone.0306312)
Supplement: Attachment — Submitted filename: Response to Reviewers - atr.docx [file pone.0306312.s004.docx]

Tacna, June 3rd, 2024

Dear Editor,

I hereby submit for your consideration the revised version of the manuscript, now titled "Public interest in palliative care in Latin America: A Google Trends analysis," for possible publication in your prestigious journal.

All co-authors have thoroughly reviewed the suggestions, comments, and annotations provided on the previous version of the manuscript, to which we received feedback on May 23rd of this year. We greatly appreciate the time spent on your review and have addressed each suggestion as follows:

1. **How did other health-related keywords change over time? A quick look at “Google Trends” shows that the relative search volumes for both “health” and “salud” have increased from 2010 to 2023. This finding may put your results into a context that you have not discussed.**

R: The terms [salud] and [health] have consistently shown a high search volume, but it wasn't until the COVID-19 pandemic that they experienced a significant peak, which has been sustained since then. On the other hand, terms related to palliative care have been on a positive trend even before the pandemic. It is worth noting that this is a great contribution, and therefore, it has been deemed appropriate to include supplementary material showing the trend of the terms [salud] and [health] in Google Trends.

2. **Palliative care has historically focused on cancer patients. This is not the case any more; patient with manifold severe chronic diseases are provided palliative care. Thus, the search term “palliative chemotherapy” may have become less representative of palliative care internet queries since the late 2010s.**

R: This is another valuable contribution; therefore, it has been deemed appropriate to add a paragraph in the discussion. This paragraph is the penultimate one of the discussions and is cited verbatim below: **<< The evaluated terms, such as "[eutanasia]", "[euthanasia]", "[muerte digna]", "[death with dignity]", "[sedación terminal]", "[terminal sedation]", "[quimioterapia paliativa]", and "[palliative chemotherapy]", did not exhibit a clear trend, potentially due to their infrequent usage. It is important to note that while palliative care has traditionally focused on cancer patients, it now encompasses individuals with various serious chronic illnesses. As a result, the terms "[quimioterapia paliativa]" and "[palliative chemotherapy]" may be less commonly used today, which could explain the low search frequency observed in our study..>>**

3. **What about searches in other South American languages than Spanish, like Quechua for the Andes, or “paliativismo,” as apparently used in Brazil? Attempts to include ethnic minorities should be visible in the analysis.**

R: This analysis with the Portuguese term [paliativismo] has been added to the supplementary

material. Likewise, it's worth noting that a precise term has not been found for the Quechua or Aymara languages.

4. **During the selected time frame, public health crises occurred in Latin America. Particularly the Zika virus outbreak and the COVID pandemic can be associated with needs for palliative care. How do they show in your analysis?**

R: This positive trend in favor of palliative care does not seem to have been influenced by the public health issues that LATAM has experienced. Accordingly, we have added the following paragraph in the discussion section: “Additionally, while the search of the terms "[salud]" and "[health]" showed an increasing trend only following the COVID-19 pandemic in 2019/2020, the terms "[paliativos/paliativo/paliativa/paliativas]" have shown a continuous rise since the 2010s. This suggests that the growing interest in palliative care is not merely a secondary effect of the increased interest in health due to the COVID-19 pandemic. Furthermore, the COVID-19 pandemic and other public health crises in Latin America, such as the Zika virus outbreak, do not appear to correlate with significant changes in the overall trend of interest in palliative care.”

5. **The correlation in figure 4 may not satisfy all statisticians…**

R: Although a positive correlation was found between the VRS and level of development, this result is inconclusive due to the small number of countries analyzed and significant exceptions. For instance, Ecuador exhibits a low level of development but a high search volume, while Argentina displays a high level of development but a low search volume. Additionally, this was added as a new paragraph in the Discussion section, which is cited verbatim below: **<<While a positive correlation between the VRS and the level of development of palliative care was observed, this finding remains inconclusive due to the limited number of countries analyzed and notable exceptions. For instance, Ecuador demonstrates a low level of development but a high search volume, whereas Argentina shows a high level of development but a low search volume.>>**

6. **In the references, “disponible en” should be translated into English.**

R: This term has been translated in the references section.
